# Are Effects of Action on Perception Real? Evidence from Transformed Movements

**DOI:** 10.1371/journal.pone.0167993

**Published:** 2016-12-15

**Authors:** Wladimir Kirsch, Benjamin Ullrich, Wilfried Kunde

**Affiliations:** Department of Psychology, University of Würzburg, Würzburg, Germany; University of Muenster, GERMANY

## Abstract

It has been argued that several reported non-visual influences on perception cannot be truly perceptual. If they were, they should affect the perception of target objects and reference objects used to express perceptual judgments, and thus cancel each other out. This reasoning presumes that non-visual manipulations impact target objects and comparison objects equally. In the present study we show that equalizing a body-related manipulation between target objects and reference objects essentially abolishes the impact of that manipulation so as it should do when that manipulation actually altered perception. Moreover, the manipulation has an impact on judgements when applied to only the target object but not to the reference object, and that impact reverses when only applied to the reference object but not to the target object. A perceptual explanation predicts this reversal, whereas explanations in terms of post-perceptual response biases or demand effects do not. Altogether these results suggest that body-related influences on perception cannot as a whole be attributed to extra-perceptual factors.

## Introduction

In numerous studies, perceptual measures proved sensitive to the experimental variations of variables related to the body and its real or potential movement (see e.g., [1], [2], for reviews). The question whether these effects reflect genuine changes in what we see (i.e., in perception) or reflect extra-perceptual influences (e.g., in judgment or in memory) recently caused intensive debates (e.g., [3–8]). Specifically, it has been argued that none of previous studies provide evidence for perceptual changes and that all of them “fall prey to only a handful of pitfalls” [9]. Here, we focused on one of these pitfalls called the El Greco-fallacy and tested the validity of this fallacy for the effect of one motor variable on space perception.

The Spanish artist El Greco painted objects strangely elongated along the vertical. It has been suggested that he did so, because he saw everything vertically stretched. This conclusion, however, has been logically refuted. If his vision was altered, so the argument goes, his percept of the canvas was as stretched as that of the motif he painted. Thereby the stretched perception of the canvas would cancel out the stretched perception of the motif. Consequently, El Greco’s paintings cannot be taken as evidence for his distorted vision. However, to conclude from this argument that distorted vision cannot be expressed in distorted paintings is a fallacy too (an El Greco fallacy fallacy, [10]). If for example, an artist actually has vertically stretched vison, but more so for distant objects (the motif) than for near objects (the canvas) his paintings might well result from his specific way of seeing.

We believe that at least some of the arguments against non-visual effects in perception reflect an El Greco fallacy fallacy. Consider a recent study by Firestone and Scholl [11]. In their Experiments 4 and 5 participants were asked to write down an ethical or unethical action from their past and to subsequently judge the brightness of the room using a numerical scale (Exp. 4) or grayscale patches (Exp. 5). Irrespective of the judgment method the room was judged as darker by participants who described unethical rather than ethical actions. The authors state that this effect cannot be perceptual–“*If stimuli look darker after recalling unethical deeds*, *the scale’s patches themselves should have looked darker too*, *and the effects should have canceled each other out*” (p 45). This inference is basically correct. However, it is correct if, and only if, the impact of unethical deeds on rooms and grey patches was exactly the same. This precondition, however, is neither self-evident nor was there any indication that it was met.

Consider, e.g., a well-known phenomenon of evaluative conditioning relating to changes in the liking of a stimulus after it has been paired with another positive or negative stimulus (see e.g., [12] for a review). From the perspective of this research it is conceivable that a certain mood state and the room in which it emerged (i.e., the target object) were associatively linked due to their co-occurrence. However, for the reference object by means of which the judgment was made (i.e., the comparison object) there is no need to assume that such an associative link to the mood state was formed because this object was absent in the mood induction phase. Note that this is consistent with what we and others have implicitly or explicitly assumed in previous studies using matching procedures (cf. e.g., [13–15]). In particular, it has been presumed that the relevance of one object to the given experimental manipulation is decisive for whether the perception of that object would be affected. This hypothesis legitimates thus using of comparable stimuli as target and reference objects and consequently competes against the classic El Greco rationale. The critical point here is that these two mutually exclusive theoretical assumptions have not been empirically contrasted by Firestone and Scholl. Thus, changes in perception of the room could, in theory, well be reflected in judgments of comparison objects, be these a numerical scale or grayscale patches. Accordingly, to conclude that the effect “cannot be perceptual” is not justified. The same argument holds for all studies discussed by the authors in favor of non-perceptual explanations in which similar stimuli were used as target and reference objects.

We do, of course, not intend to state that the original conclusion that the effect (of ethical vs. unethical deeds) is due to a response or judgment bias is wrong. Rather, we point to the fact that finding an effect using similar stimuli as target and reference objects does not imply that this effect cannot be related to changes in objects’ appearance (cf. also, e.g., the research on attention and appearance, [16], [17]). Reasoning otherwise, as was done previously, is logically flawed. This issue can only be empirically solved. For example, participants could be asked to write down ethical or unethical actions on gray patches (ideally in a dark room) and to move to another room. Then, similar gray patches could be used as reference objects to judge the lightness of the second room. If the effect is due to non-perceptual factors, the original effect should be observed (i.e., brighter judgments for the ethical condition). In contrast, a perceptual explanation would predict a reversal of the original effect because now the perception of the reference and not of the target object should change.

In more general terms thus, applying the El Greco fallacy correctly presumes that an effect of interest affects both, target objects and reference objects to exactly the same extent. There is one study in which Firestone and Scholl [11] tried to keep constant possible action related effects on target and reference objects. In their Experiment 2 participants held a rod across the body, and were asked to judge the width of a target aperture. Other participants did the same without a rod. These judgments were collected by adjusting the width of a reference aperture. The supposed motor influence that originates from the rod acting as an obstacle was conceivably kept constant because participants imagined walking through both, the target aperture and the reference aperture. Still, participants with a rod judged apertures to be narrower, although the perceptual shrinking of both apertures should be the same. Consequently, this result is inconsistent with a perceptual effect, and suggests instead extra-perceptual causes such as response bias or demand effects. Note that this is the only empirical basis for the one of core arguments that visual perception is immune to action or other non-visual influences. Given this far reaching conclusion we believe that more evidence is necessary.

To test the generality of the El Greco fallacy we applied it to a body-related influence on perception, which we describe in more detail in the introduction of Experiment 1. We first start with demonstrating that a motor variable impacts perceptual judgments when applied exclusively to a target object but not to a reference object used to express these judgements. Moreover this impact reverses when applied to the reference object but not to the target object (Experiment 1). A perceptual explanation predicts this reversal, whereas explanations in terms of response biases or demand effects do not. Finally we show that parallelizing the motor manipulation between target objects and reference objects almost completely abolishes the impact of that manipulation so as it should do, according to the El Greco logic, when that manipulation primarily altered perception (Experiment 2). Altogether we thus claim to show body-related effects on perception are hard to reveal when they encompass both, target and reference objects, so as the El Greco fallacy suggests. At the same time we claim that such effects are real nevertheless as revealed by their selective impact on either target or reference objects.

## Experiment 1

This study is part of a larger project in which we examine influences of action properties on space perception in grasping space (e.g., [13], [14], [18]). Here we focused on the impact of action transformation on size perception. Action transformation means that the natural movements of the fingers are translated into altered movements of visual feedback, which commonly occurs when using tools (e.g., [19]). Transformations can be of such a kind that certain finger movements produce larger movements of visual feedback (gain > 1) or smaller movements (gain < 1). Large gains can be construed as an increase of action capability because visual movements become possible (in terms of amplitude or speed) which were not possible with natural finger movements or gains smaller than 1. In other words, large gains can be assumed to increase the actual action ability of the hand to manipulate an external object as compared with small gains.

**Fig 1:**
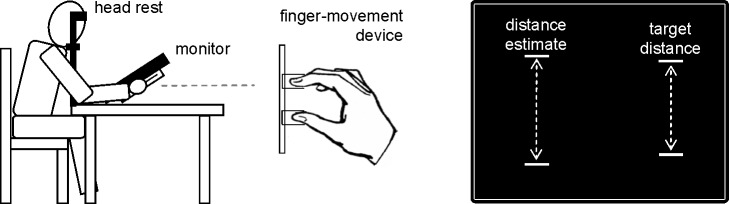
Apparatus (left and middle parts) and critical visual stimuli (right part) used in Experiments 1 and 2.

Following the common approach of research on action-specific perception we first aimed to demonstrate that an experimental variation of gain and thus of current action capability affects visual distance judgments in Experiment 1a. Participants manipulated a movement device behind a monitor with the thumb and index finger of the right hand (see Fig 1). The finger movements were transformed into movements of two visual bars on the right side of the screen. After a certain target distance between the bars was reached, participants judged that distance. This was done by matching the distance of two comparison bars presented on the left side of the screen. The critical experimental variation concerned the gain of the transformation of finger movements to movements of the bars, which was either smaller than 1 or larger than 1. In other words the transformation either decreased or increased the actual action capability of the hand. This manipulation was applicable only to the target object, whereas it was inapplicable to the comparison object.

Typically, an increase in action capability is associated with a decrease in the estimated size of objects to which action is related (e.g., [20–22]; cf. also [23], [6], [15], for related findings in distance perception). For example, a magnification of the size of the hand proved to decrease the size estimates related to graspable objects [21]. Consequently, increasing the gain between finger movements and the movements of visual objects served as movement feedback should reduce the perceived distance between those objects. We thus predicted a kind of contrast effect in Experiment 1a: smaller judgments of object distance with large action capability (large gain) than with small action capability (small gain). It should also be mentioned that larger gains went along with smaller hand openings as compared with smaller gains. Accordingly, the same effect can also be potentially predicted based on cross-modal research (e.g., [24]; cf. also [25]): sensory integration of the posture with the visual object information can here be expressed in a bias towards the haptic signal (see also [26]). Please note, however, that the logic in respect to the present question of interest is clear and straight forward irrespective of the exact origin of the expected effect. In particular, the critical experimental variation can be considered as either related to the current body state (cross modal hypothesis) and / or to its potential action (action capability hypothesis). In either case, thus, a body-related variable is assumed to affect visual perception and this is questioned by the El Greco argument.

Reasoning this way is fully consistent with the research on action-specific perception where the impacts of morphological and action-related variables are discussed within a common theoretical framework (e.g., [1]). Firestone and Scholl [9], however, recently and paradoxically agreed that several types of action-related variables (including effective body size) cannot affect perception due to several mostly theoretic reasons but cross-modal (i.e., including visual-haptic) processes potentially can. We strongly disagree with this limited view and see neither convincing theoretical nor empirical arguments for such a distinction.

Given we observed the predicted pattern, would that really reflect altered perception? Participants perhaps merely responded in accordance with their guess of the experiments’ purpose as presumed by Firestone and Scholl [11], hence a response bias. Experiment 1b was done to contrast these alternatives. We shifted the transformation of the fingers movements from the target distance to the comparison object. Participants were asked to judge the same distances as in Experiment 1a. They did this, however, by manipulating the finger movement device. That is, the movements of the fingers were now transformed into movements of the bars which were used as matching stimuli. The target object was not manipulable. If the large gain was still associated with smaller distance estimates than the small gain, then non-perceptual processes would account for the effect of the gain. According to the perceptual explanation, however, the original effect should reverse in Experiment 1b. If higher action capability (i.e. large gain) decreases the perceived distance between the manipulated bars then the distance by means of which the target distance is judged should now appear smaller for the large gain as compared to the small gain. Because the task is to equalize the estimate with the target distance participants should now produce larger estimates for the large gain (in order to compensate for the distortion of the estimate).

### Methods

#### Ethics Statement

All participants volunteered and provided written informed consent. The study was conducted in accordance with German Psychological Society (DGPs) ethical guidelines (2004, CIII). This research was also reviewed and approved by the German Research Council (DFG, project number KI 1620/1-2) which did not require Institutional Review Board approval. No identifying information was obtained from the participants for the purpose of the study apart from their age and handedness. Moreover, written informed consents (which were signed by the participants) informed the participants that their data will be anonymously (i.e. without access to their names) saved, analyzed and published. Accordingly, the present article does not include any identifying, or potentially identifying, information to which participants did not consent.

#### Participants

Twenty-six right-handed participants participated in Experiment 1. Participants received monetary compensation or course credit for their participation. The sample size was chosen on the basis of our experience with similar setups and related research questions (cf., e.g., [14]). Sample sizes amounting to about 24 participants have proven appropriate in the past to demonstrate substantial systematics. One participant aborted the experiment due to problems with the handling of the apparatus. Judgment errors of another participant were very large in some conditions and considerably deviated from those of other participants (partly above 4 cm on average). The data of these participants were not included in the analyses. The final sample included six males and eighteen females (*M*_*age*_ = 26 years, *SD* = 8).

#### Apparatus and stimuli

The main apparatus consisted of a 19’ monitor (Fujitsu Siemens P19-1) and a finger movement device mounted behind the monitor (see Fig 1). This apparatus was positioned on a table at an angle of about 40 degrees. One pixel (px) of the monitor was 0.294 mm in size. The finger movement device was manipulated by the index finger and the thumb of the right hand. The fingers were placed on U-shaped metal plates with a “horizontal” distance of about 1.7 cm between both sites (that is fingers were not completely fixed). Both metal plates were interlocked so that moving one plate / finger resulted in a mirror-symmetric movement of the second plate / finger.

Visual stimuli were gray bars about 7 mm in length and 1.2 mm in width presented on a black background. One pair of bars that was defined as target distance was always shown on the right side of the screen (about 10 cm in respect to the right edge) approximately above the fingers. The other pair of bars by means of which target estimates were made were always on the left side of the screen (about 12 cm in respect to the left edge and about 14 cm apart from the target distance).

Participants were seated at a distance of approximately 41 cm from the screen with their head supported by a combined chin-and-forehead rest. Moreover, headphones were used for the presentation of acoustic signals and to minimize external noise. A conventional computer mouse lay on the table left to the main apparatus and was manipulated by the left hand in Experiment 1a.

#### Procedure and Design

Experiment 1 consisted of two parts, namely of Experiment 1a and Experiment 1b. Each participant performed both experiments within one single session. The order of the Experiments 1a and 1b was counterbalanced across the participants.

#### Experiment 1a

**Fig 2:**
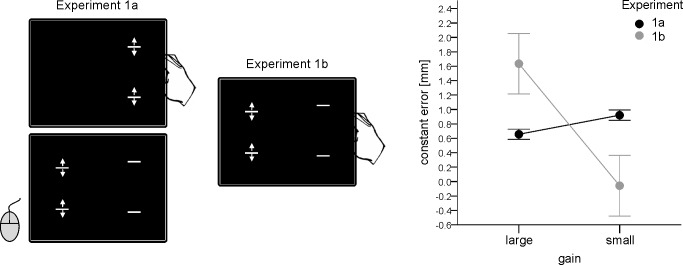
Schematic illustration of the main trial events (left and middle part) and main results (right) in Experiments 1a and 1b. Error bars indicate within-participants confidence intervals (95%) computed according to [27]. When the gain manipulation was applied to the to be judged object (Exp. 1a) participants judged distances as larger with the small than with the large gain. In contrast, when the same gain manipulation was applied to the object by means of which the judgment was made (Exp.1b) distances were judged as larger with large than with the small gain.

The main trial events of Experiment 1a are shown in Fig 2 (left part). Participants moved their fingers of the right hand inserted into the finger movement device until they heard a clicking noise. Participants were instructed to maintain a finger position at which the noise was nonstop presented for 1.5 sec and to perform corrective movements when the noise disappeared. These movements were accompanied by the movements of the two bars presented on the right side of the screen. The bars always moved in the same direction as the fingers (i.e., to each other or away from each other). The noise appeared when the distance between the bars, i.e. the target distance, reached 34, 40 or 46 mm. The initial distance between the bars corresponded to the current distance between the fingers modified by the current gain factor. That is the starting position of the fingers varied trial by trial and corresponded to their end position in the previous trial (see below).

After the bars were positioned at the right distance (and following a period of 1.5 sec in which the noise was presented) an additional pair of bars appeared at the left side of the screen. The initial distance between those bars randomly amounted either 50 or 150% of the target distance. The task was to adjust the distance between the bars on the left side so that it corresponded to the distance on the right side (which was permanently visible during the adjustment procedure). This judgment was made per pressing buttons of a computer mouse. Pressing the left / right button led to an increase / a decrease of the distance. The judgment was confirmed by pressing the middle mouse button (scroll wheel).

When participants changed the fingers’ posture of their right hand during the judgments, or when the left or the right mouse buttons were pressed before the bars on the left side appeared, or when the middle mouse button was pressed before an estimate was made, an error feedback was presented and the trial was repeated.

The critical experimental variation was related to the transformation of the finger movements to the movements of the bars. The distance between the fingers was multiplied either by the factor of 2/3 or by 2. That is, in one condition the finger distance was always larger than the distance between the bars (with a relation of 1.5:1, small gain). In another condition, the finger distance was smaller than the distance between the bars (0.5:1, large gain). Note that for a given target distance the visual input was identical for both gain conditions during the judgment procedure.

The main experiment included 3 blocks of trials with 48 trials each (8 repetitions of each condition in each block). The order of conditions was random (with the constraint that no immediate repetitions of the same condition were possible and all conditions should be completed before a next repetition was possible). Before the main experiment started participants performed 12 practice trials which were not included in the analyses.

#### Experiment 1b

In Experiment 1b the target distance appeared together with the bars used for the estimate. The task was (as in Exp. 1a) to judge the target distance presented on the right side of the screen by the adjustment of the bars presented on the left side of the screen. The judgment was made by manipulating the finger movement device with the right hand and was confirmed by pressing the scroll wheel with the left hand (see Fig 2, middle part). When finger movements were transferred into the movements of the bars, the same gains were applied as in Experiment 1a (i.e., 2/3 or 2). All other aspects of the design were identical to those of Experiment 1a.

#### Data preprocessing

A difference score was computed between the actual and the estimated distance for each trial (constant error hereafter). By definition, positive values reflect overestimation, negative values reflect underestimation. Trials in which the constant error was below the first quartile minus 1.5 times the interquartile range or above the third quartile plus 1.5 times the interquartile range as computed for each participant, each experiment, each target distance and gain condition were considered as outlier and were excluded from analysis. Overall, 97.9% of trials entered the analyses (The raw data can be obtained in S1 File).

### Results and discussion

The main results are shown in Fig 2 (right part). As predicted, a small gain was associated with an increase in judged distance when judgements were made per mouse buttons (Exp. 1a). For the judgments performed with the finger movement device an inverse pattern was evident (Exp. 1b). We performed an analysis of variance (ANOVA) including target distance, gain and judgment mode (i.e., Exp. 1a and 1b) as within-participants factors. The critical interaction between gain and judgment mode was significant with *F*(1, 23) = 22.76, *p* < .001, *η*_*p*_^*2*^ = .497. In this analysis, an interaction between all of the factors was also significant, *F*(2, 46) = 4.42, *p* = .017, *η*_*p*_^*2*^ = .161, additionally to the main effects of gain, *F*(1, 23) = 11.93, *p* = .002, *η*_*p*_^*2*^ = .342, and distance, *F*(2, 46) = 15.54, *p* < .001, *η*_*p*_^*2*^ = .403 (other *p* > .346). An increase in target distance resulted in a decrease of the constant error. The observed three-way interaction indicated an increase of the impact of gain with an increase in target distance for both judgment modes (see S1 Table for mean values).

Follow up analyses (ANOVAs) including target distance and gain as within-participants factors and performed for each experiment separately revealed significant main effects for the factor gain for both judgment modes, *F*(1, 23) = 14.86, *p* = .001, *η*_*p*_^*2*^ = .392 (Exp. 1a), and *F*(1, 23) = 17.37, *p* < .001, *η*_*p*_^*2*^ = .430 (Exp. 1b). The main effect of target distance was also significant, *F*(2, 46) = 9.48, *p* < .001, *η*_*p*_^*2*^ = .292 (Exp. 1a), and *F*(2, 46) = 9.69, *p* < .001, *η*_*p*_^*2*^ = .296 (Exp. 1b). No significant interactions were observed (*ps* > .055, but see above).

To conclude, these results suggest that the impact of the implemented manipulation of gain on distance estimates is perceptual rather than non-perceptual in nature.

## Experiment 2

The results of Experiment 1 were clear cut. Nevertheless, some caveats remain. For example, judgments made using the movement device in Experiment 1b could be affected by a response bias specific to this judgment mode. This could in theory replace the response bias of interest possibly observed in Experiment 1a (larger judgments with small gains). Thus, additional control conditions appeared necessary.

The rationale of Experiment 2 was similar to that of Experiment 2 in [11]. Hence we attempted to equalize the motor impact on perception between target objects and reference objects. We did so by making both types of stimuli (i.e., bars used for target distance and for distance estimate) equally irrelevant to the “grasping” posture in Experiment 2a and equally relevant in Experiment 2b. That is, finger movements of the right hand neither affected the target distance nor the distance estimate or they affected both. We pursued a very conservative approach and changed the original setups only slightly. Participants moved an additional pair of bars in Experiment 2a before they judged the target distance by button presses. In Experiment 2b, they “produced” the target distance initially by means of the movement device (as in Experiment 1a) and then judged that distance using the movement device again.

The predictions were straightforward. The perceptual hypothesis predicts that the effects observed in Experiment 1 should be reduced or even disappear in Experiment 2. If however, participants simply judge distances as smaller with larger gains when they press buttons and as larger with larger gains when they use the movement device, there appears to be no reason why they should not do so in Experiment 2 to the same extent as in Experiment 1.

### Methods

#### Participants

Twenty-five right-handed participants participated in Experiment 2. None of them participated in Experiment 1. Participants gave their informed consent for the procedures and received monetary compensation or course credit for their participation. One participant aborted the experiments due to problems with the task. The data of this participant was not included in the analyses. The final sample included eight males and sixteen females in Experiment 2 (*M*_*age*_ = 27 years, *SD* = 8).

#### Apparatus and stimuli

Apparatus and stimuli were the same as in Experiment 1. Additionally, two yellow bars were used in Experiment 2a which were of the same size as the gray bars used in Experiment 1. The horizontal position of these bars was in the middle between the gray bars used as target distance and those used for the estimate.

#### Procedure and Design

Procedure and design of Experiment 2 were similar to the procedure and design of Experiment 1. Thus, we only describe the main differences below.

#### Experiment 2a

**Fig 3:**
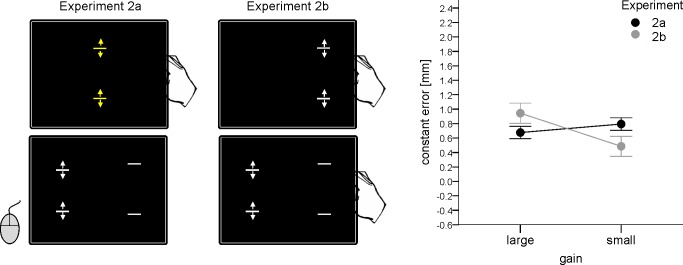
Schematic illustration of the main trial events (left and middle part) and main results (right) in Experiments 2a and 2b. Error bars indicate within-participants confidence intervals (95%) computed according to [27]. When the gain manipulation was applied to an object being neither target nor comparison object (Exp. 2a) no significant difference between the gain conditions was observed. When the gain manipulation was applied to the to be judged object as well as to the object by means of which the judgment was made (Exp.2b) distances were judged as larger with large than with the small gain as in Exp. 1b, however, to a much lesser extent (see Fig 2).

The main trial events of Experiment 2a are shown in Fig 3 (left part). At the beginning of each trial two yellow bars appeared. The distance between these bars corresponded to the actual finger distance multiplied by the gain factor. By moving the fingers participants searched for the clicking noise as in Experiment 1a. The noise was presented when the distance between the bars reached 34, 40 or 46 mm. After the bars were positioned at the right distance and following a period of 1.5 sec in which the noise was presented the yellow bars disappeared and two pairs of gray bars appeared (to the left and to the right of the middle bars at the same horizontal positions as in Exp. 1). The distance between the bars presented on the right side of the screen reflected again the target distance and corresponded to the end distance of the yellow bars. The initial distance of the bars shown on the left side amounted either 50 or 150% of the target distance as in Experiment 1a. Participants were again asked to judge the target distance by means of the adjustment of the left distance. Thus, the only critical difference between Experiment 1a and Experiment 2a was that participants manipulated stimuli being neither judged nor used as means for judgments before they estimated a certain distance.

#### Experiment 2b

The initial phase of Experiment 2b was identical with the initial phase of Experiment 1a (see Fig 3, middle part). That is, participants first moved their fingers until a certain target distance was reached. Then, they were asked to move the fingers back and forth again until an additional pair of bars appeared on the left side of the screen. These bars appeared when the finger distance corresponded to either 50 or 150% of the target distance. Then, the participants judged the target distance as in Experiment 1b (i.e., by finger movements). In each trial the transformation of the finger movements into the movements of the bars was always the same (i.e., the gain did not change within a single trial).

#### Data preprocessing

Data preprocessing was performed in the same way as in Experiment 1. Overall, 98.4% of trials entered the analyses (The raw data can be obtained in S1 File).

### Results and discussion

As shown in Fig 3 (right part), the pattern of main results observed in Experiment 1 is also present in Experiment 2, being however far less pronounced. An analysis including the data of Experiments 2a and 2b and gain, distance and judgment mode as factors revealed significant main effects of gain, *F*(1, 23) = 5.38, *p* = .030, *η*_*p*_^*2*^ = .190, and distance, *F*(2, 46) = 18.03, *p* < .001, *η*_*p*_^*2*^ = .439, and significant interactions between gain and judgment mode, *F*(1, 23) = 11.71, *p* = .002, *η*_*p*_^*2*^ = .337, and between judgment mode and distance, *F*(2, 46) = 6.37, *p* = .004, *η*_*p*_^*2*^ = .217 (other *p* > .269). An increase in target distance resulted in a decrease of the constant error (see also Exp. 1) and this impact of distance was somewhat smaller in Exp. 2a than in Exp. 2b (cf. S2 Table). The gain x judgment mode interaction suggested differential impacts of gain in both experiments which, however, was basically due to an effect of gain in Exp. 2b, but not in Exp. 2a (see below).

In separate analyses of each experiment, a significant impact of the factor gain was evident in the judgments made with the finger movement device (Exp. 2b), *F*(1, 23) = 11.65, *p* = .002, *η*_*p*_^*2*^ = .336. When judgments were made per mouse buttons, in contrast, the varying gain did not affect the constant error substantially, *F*(1, 23) = 2.01, *p* = .170, *η*_*p*_^*2*^ = .080 (Exp. 2a). The main effect of distance was also significant in these analyses, *F*(2, 46) = 8.44, *p* = .001, *η*_*p*_^*2*^ = .268, and *F*(2, 46) = 19.28, *p* < .001, *η*_*p*_^*2*^ = .456, for judgements made with the mouse and movement device respectively (see S2 Table for mean values). No significant interactions were observed (*ps* > .323).

#### Between-experiment analyses

To compare the results of Experiment 1 and Experiment 2 we initially performed an analysis (ANOVA) including the data of all conditions. That is, gain, target distance, and judgment mode (i.e., experiment-type “a” or “b”) served as within-participants factors whereas experiment (i.e., 1 or 2) served as a between-participants factor. This analysis revealed a significant interaction between gain, judgment mode and experiment, *F*(1, 46) = 9.70, *p* = .003, *η*_*p*_^*2*^ = .174, indicating a significant decrease of the impact of gain in Exp. 2 as compared with Exp. 1.

This analysis also revealed significant main effects of gain, *F*(1, 46) = 16.25, *p* < .001, *η*_*p*_^*2*^ = .261, and distance, *F*(2, 92) = 33.34, *p* < .001, *η*_*p*_^*2*^ = .420, and significant interactions between gain and experiment, *F*(1, 46) = 6.14, *p* = .017, *η*_*p*_^*2*^ = .118, between gain and judgment mode, *F*(1, 46) = 32.64, *p* < .001, *η*_*p*_^*2*^ = .415, between distance and judgment mode, *F*(2, 92) = 4.96, *p* = .009, *η*_*p*_^*2*^ = .097, and between gain, distance and judgment mode, *F*(2, 92) = 5.68, *p* = .005, *η*_*p*_^*2*^ = .110 (all other *p* > .269). The mean difference between the gain conditions was overall smaller in Exp. 2 than in Exp. 1. There was a smaller impact of distance in the judgments with the mouse (i.e., in Exp. 1a and 2a) than in the judgments with the movement devise (i.e., in Exp. 1b and 2b) and there was an increase of the impact of gain with an increase in target distance for both judgement modes (cf. also the results of previous analyses).

We also performed an ANOVA for each judgment mode separately including target distance and gain as within-participants factors and experiment as a between-participants factor. The critical interaction between the factors gain and experiment did not reach significance for the mouse judgments (i.e., for Exp. 1a and 2a), *F*(1, 46) = 1.86, *p* = .179, *η*_*p*_^*2*^ = .039, but was significant for the finger movement device (i.e., for Exp. 1b and 2b), *F*(1, 46) = 8.32, *p* = .006, *η*_*p*_^*2*^ = .153. The factors gain and distance were significant with *F*(1, 46) = 12.59, *p* = .001, *η*_*p*_^*2*^ = .215, and *F*(2, 92) = 17.21, *p* < .001, *η*_*p*_^*2*^ = .272, in the analysis of mouse judgments, and with *F*(1, 46) = 25.29, *p* < .001, *η*_*p*_^*2*^ = .355, and *F*(2, 92) = 26.76, *p* < .001, *η*_*p*_^*2*^ = .368, for the finger movement devise. A significant interaction was observed between gain and distance for the finger movement device, *F*(2, 92) = 4.18, *p* = .018, *η*_*p*_^*2*^ = .083 (all other *p* > .111). This interaction indicated an increase in gain effect with an increase in distance (see also above and S1 and S2 Tables). These results indicate that the impact of gain in Experiment 1 was substantially reduced in Experiment 2 for the judgments performed using the finger movement device. Given that the gain effect was significant in Exp. 1a but not in Exp. 2a, the same holds for the mouse judgments. However, since the direct comparison between both experiments did not reach significance, this conclusion should be considered with caution.

In spite of this, the results as a whole suggest that a substantial portion of the impact of gain manipulation on distance estimates observed in Experiment 1 is perceptual. It is also notable that even the residual impact of the gain observed in Experiment 2b could also be perceptual rather than non-perceptual. This can be expected because the manipulation of the target distance by the fingers always occurred before the manipulation of the reference stimuli. Thus, an impact of the gain during the estimate can be assumed to be larger for the reference stimuli than for the target stimuli. Admittedly, this residual effect observed in Experiment 2b (as well as the descriptive trend in Experiment 2a) could also be related to a response bias specific to the mode of judgments.

## General discussion

Can our body affect what we perceive? A methodical argument has been raised against studies which claimed to have shown such perceptual changes. The problem identified is that possible changes in perception cannot be revealed by judgement procedures which use similar target objects and reference objects, because motor influences should change the perception of both types of objects equally and thus cancel each other out [9], [11]. We took up this rationale in the present study and examined how the impact of a body-related manipulation changes depending on whether it relates to the target object, the comparison object, or to both. The results indicated a dependence of the effect’s direction and magnitude on the relation of the manipulation to the stimulus used either as target or as comparison.

In particular, larger movement-feedback gain was associated with smaller distance estimates when the to be judged distance was manipulated by the observer (Exp. 1a). In contrast, larger movement-feedback gain led to larger estimates when the stimuli by means of which the judgment was made were manually manipulated (Exp. 1b). These effects were substantially reduced when finger movements were related neither to the target nor to the reference stimuli (Exp. 2a) or when finger movements were related to both types of stimuli (Exp. 2b). The results suggest that body-related signals biased visual perception of objects to which these signals were related.

Our conclusion of a perceptual origin stands in contrast to the conclusions of Firestone and Scholl [11], who favored an extra-perceptual explanation of their action-related impact on judgment. It is true that we studied a different top-down influence on perception. Hence, it might be that the action-related impact studied by these authors was mediated by response bias, whereas the one studied by us was not. Also we do not want to exclude the possibility that some non-perceptual factors might explain some of the top-down effects reported. However, at present there is no empirical evidence for the assumption that any reported top-down effects except for the one tested by Firestone and Scholl in Experiment 2 [11] fall prey to the pitfall of El Greco fallacy (i.e., are due to non-perceptual factors). This considerably limits the conclusions of Firestone and Scholl [9], [11], but does not diminish their overall contribution to the current debate on putative non-visual effects on perception. In contrast, we appreciate the introduction of a valuable and promising paradigm that we adopted in the present study and that can help to refine and to test theoretical accounts and ensuing predictions in future studies.

Body-related effects observed in perceptual judgments are often explained by a kind of direct scaling of initial visual input in motor units (e.g., [1]). However, many reported effects, including the present one, are rather small in magnitude and are not proportional to the magnitude of the experimentally introduced body-related changes (cf. also [7]). This fact cannot be readily explained by a direct scaling approach. In one of our recent manuscripts we tackle this issue and suggest that body or action-related effects observed in visual judgments can, in principle, be explained by well-known mechanisms of multisensory integration [28]. In a series of experiments using a kind of virtual grasping and reaching tasks we observed that visual and somatic information is combined in judgments of the objects being grasped or reached as well as in judgments of body states (grasping posture or distance covered by the hand) even when the body and visual signals were spatially clearly separated. Here, multisensory integration was observed under conditions similar to those often used to demonstrate body or action-related effects on perception where a distant object is manipulated by motor actions. This finding suggests that integration of redundant sensory signals is not restricted to their spatial proximity when there is a systematic and predictable relation between them (see also [25], [29–31]). Thus, in principle, models of multisensory integration could be applied to several action-specific effects. For example, a weighed sensory sampling approach (e.g., [32]) could explain why the effects, including the present one, are often small in magnitude or even undetectable at all [33]. In particular, the effect magnitude should critically depend on the reliability (i.e., quality) of visual and body-related information and should increase with a decrease in reliability of visual and with an increase in reliability of body-related signals. Since visual information can be assumed to be more reliable in many usual setups the impact of the body in the whole estimate would be rather small.

The reader might wonder how this can be related to other rather “classical” effects reported so far. Consider, e.g., the finding that hills are judged steeper when wearing a heavy backpack [34]. Now assume that there is a certain variable related to the imagined (or simulated) body perception associated with the walking up a hill which is taken into account in the visual perception of that hill (cf. e.g., [35]). Combining both would not be implausible from a multisensory perspective since an integrated estimate usually achieves a higher precision that the unimodal signals alone. In this view, this finding is not surprising and resembles the observation that the visual perception of slant in grasping space is attracted by the slant experienced through haptics [36].

It is worth mentioning that the current debate about whether top-down effects are perceptual or not might appear artificial for researchers from other perceptual domains. In particular, the Bayesian approach to perception which has received much attention relies on the assumption that decision processes are an integral part of perception (e.g., [37], [38]). In other words, there is no percept without decision here. The terms such as judgment or response biases as used in the present manuscript and related works, however, mean processes which are not a part of the current percept. This is not incompatible with a Bayesian view where “prior knowledge” or “inference” can be assumed to be related to low-level automatic perceptual processes with no mandatory access to higher cognition [39].

We want to conclude by pointing to a general problem of the El Greco fallacy. Even if one accepts that El Greco’s paintings cannot be taken as evidence for his distorted vision, one cannot conclude inversely that his vision was actually intact. We simply cannot know from that. He might well have had distorted vision of both, the canvas and the rest of the world. However, other procedures were necessary to reveal that, such as demonstrating that he saw vertical rectangles where normal observers saw squares. Analogously, wearing a rod across one’s body might well affect perception, such that *both*, target apertures and comparison apertures look narrower, so that the equally affected perception cannot show up under such conditions. But still it is perfectly possible that target apertures and comparison apertures both actually *do* look narrower. Only other procedures were necessary to reveal that, such as using still other comparison apertures to which the proposed motor influence does not extend. This we believe is an empirical endeavor. Such endeavors might reveal that many supposed non-visual effects on perception are in fact absent where they should be absent, and present where they should be present.

## Supporting Information

S1 File Raw data of all experiments. (SAV)

S1 Table Mean constant judgment error in Experiment 1a and Experiment 1b [in mm]. Note, standard deviations are in parentheses.
(DOCX)

S2 Table Mean constant judgment error in Experiment 2a and Experiment 2b [in mm]. Note, standard deviations are in parentheses.
(DOCX)
