# Supplementary material for: Are Effects of Action on Perception Real? Evidence from Transformed Movements
Source: PLoS One. 2016 Dec 15;11(12):e0167993. doi: 10.1371/journal.pone.0167993 (PMC5158014; doi:10.1371/journal.pone.0167993)
Supplement: S2 Table — Note, standard deviations are in parentheses. (DOCX) [file pone.0167993.s003.docx]

| **Exp. 2a** |  | *Target distance [in mm]* | | |
| --- | --- | --- | --- | --- |
|  |  | *34* | *40* | *46* |
| *gain* | *large* | 0.9 (1.2) | 0.8 (1.4) | 0.4 (1.3) |
|  | *small* | 0.9 (1.3) | 0.9 (1.3) | 0.6 (1.5) |
| **Exp. 2b** |  | *Target distance [in mm]* | | |
|  |  | *34* | *40* | *46* |
| *gain* | *large* | 1.3 (1.5) | 0.9 (1.9) | 0.6 (1.7) |
|  | *small* | 1.0 (1.4) | 0.5 (1.6) | 0.0 (1.5) |
